# Role of Topical Insulin in Severe Ocular Graft-Versus-Host Disease With Acute Bilateral Perforation

**DOI:** 10.7759/cureus.85468

**Published:** 2025-06-06

**Authors:** Pedro Moreira Martins, Jorge Costa, Carolina Madeira, Ricardo Machado Soares

**Affiliations:** 1 Ophthalmology, Unidade Local de Saúde de Gaia e Espinho, Porto, PRT

**Keywords:** corneal perforation, dry eye syndrome, ocular graft vs host disease, refractory corneal ulcer, topical insulin therapy

## Abstract

Graft-versus-host disease (GVHD) hinders the prognosis of patients undergoing allogeneic hematopoietic stem cell transplantation (allo-HSCT). Ocular GVHD (oGVHD) presents as dry eye syndrome (DES) and may progress to corneal perforation. We report a case of acute bilateral corneal perforation in a 42-year-old male with chronic GVHD. The patient presented with mild pain in his right eye (OD [oculus dexter]) and was diagnosed with corneal perforation. Cyanoacrylate glue and amniotic membrane were acutely applied in OD. However, despite appropriate medical treatment, he later developed a refractory, painless central ulcer. During follow-up, the patient also experienced an unexpected, asymptomatic, acute perforation in the left eye. Due to progressive deterioration of the OD ulcer, topical insulin was initiated, resulting in rapid re-epithelialization and control of oGVHD.

This is the second known report of topical insulin therapy in ocular GVHD and the first to document rapid epithelial healing following bilateral spontaneous corneal perforation. It highlights how oGVHD may result in corneal perforation despite adequate treatment and the role of topical insulin in promoting epithelial healing and stabilizing the ocular surface.

## Introduction

Allogeneic hematopoietic stem cell transplantation (allo-HSCT) is an effective therapeutic approach for several hematological disorders. However, its success is frequently compromised by the onset of post-transplant graft-versus-host disease (GVHD), a condition that increases patient morbidity and mortality [1]. Chronic GVHD (cGVHD) is an immune-mediated disorder that commonly involves ocular manifestations (oGVHD), most notably presenting as dry eye syndrome (DES). In rare cases, severe ocular complications such as persistent epithelial defects (PEDs) and corneal ulcers refractory to conventional treatments may occur, potentially leading to corneal perforation [1,2].

Topical insulin has recently emerged as a potential treatment for neurotrophic corneal ulcers [3,4]. However, the full scope of its utility is still under investigation, and its use in GVHD has been documented in only one prior report [5]. This paper presents a case of refractory oGVHD complicated by acute, spontaneous bilateral corneal perforation. Following the treatment of the perforation, the patient’s oGVHD-related DES and subsequent corneal ulcer were successfully managed with topical insulin.

## Case presentation

We report the case of a 42-year-old male who presented to the emergency department with complaints of mild discomfort and tearing in his right eye (OD [oculus dexter]) persisting for three days. The patient was followed in another center for cGVHD with ocular, hepatic, and pulmonary involvement following peripheral blood allo-HSCT from a human leukocyte antigen-matched sibling for myelodysplastic syndrome and paroxysmal nocturnal hemoglobinuria, five years prior. At the time of ocular evaluation, both paroxysmal nocturnal hemoglobinuria and myelodysplastic syndrome were clinically stable, without evidence of active disease or relapse. Current medication included prednisolone 20 mg daily, cyclosporine 50 mg daily, and ruxolitinib 30 mg daily, with poor compliance. According to his medical history, the patient’s oGVHD was characterized by refractory moderate-to-severe DES, PEDs, and corneal ulcers, which had been treated with various topical medications (lubricants, corticosteroids, antibiotics, cyclosporine) and therapeutic contact lenses (CLs). 

Upon examination, the patient’s best-corrected visual acuity (BCVA) was light perception in the OD and 20/400 in the left eye (OS [oculus sinister]). The OD exhibited mild conjunctival hyperaemia, a 1-mm translucent central corneal perforation, and complete shallowing of the anterior chamber with iridocorneal contact. The OS displayed corneal leukomas with mild keratitis. Cyanoacrylate glue was acutely applied to the perforation site, along with a bandage CL and antibiotics. The following day, the anterior chamber depth was restored, and the patient was scheduled for an inlay amniotic membrane graft. Additionally, following consultation with the assisting oncologist, systemic prednisolone was increased to 40 mg daily. One month after surgery, the inlay graft had successfully integrated with the corneal stroma, and the patient was maintained on aggressive lubrication and topical fluorometholone in both eyes due to persistent DES symptoms. Although the Ocular Surface Disease Index (OSDI) questionnaire has not been validated for patients with amniotic membrane grafts or corneal perforations, as the reduced visual acuity may confound assessment, it was nevertheless performed in this case, corroborating the severity of DES as indicated by the severity score of 83.3. Six out of the 12 questions were used in the analysis (score: 22), as questions 4, 5, 6, 7, 8, and 9, which pertain to visual acuity, were left unanswered [6]. Bilateral corneal sensitivity was absent, as determined by testing with a sterile cotton thread. The corneal thickness at the thinnest point, measured by anterior segment optical coherence tomography (AS-OCT), was 209 µm, and the tear break-up time was 2 seconds. 

**Figure 1:**
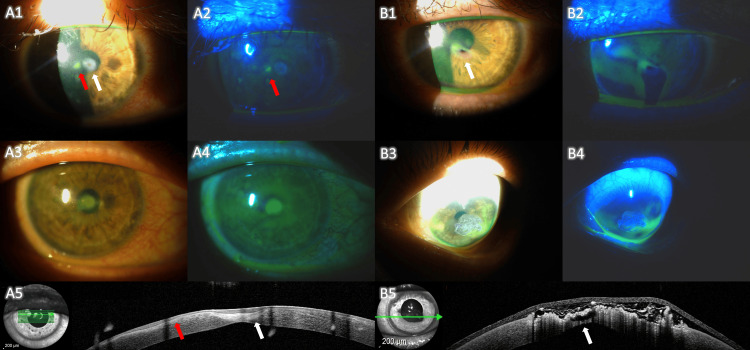
Clinical images of the right (A1-A5) and left (B1-B5) eyes of the patient, with and without cobalt-blue filter and fluorescein stain. A1 and A2: Right eye, four months post-inlay amniotic membrane graft surgery. The integration of the amniotic membrane at the site of perforation is visible (white arrow), alongside a paracentral epithelial defect with mild stromal ulceration (red arrow). B1 and B2: Spontaneous perforation of the left eye, demonstrating a translucent epithelial defect (white arrow) and a positive Seidel test. B3 and B4: Left eye following cyanoacrylate glue application. B5: Anterior segment optical coherence tomography (OCT) of the left eye, highlighting the cyanoacrylate glue covering the probable site of perforation (white arrow). A3 and A4: Right eye after two months of topical insulin treatment, showing significant improvement in punctate keratitis and fluorescein pooling in the previous perforation zone. A5: Anterior segment OCT of the right eye, demonstrating the integration of the amniotic membrane graft (white arrow) and re-epithelialization of the paracentral corneal ulcer (red arrow).

Four months post-surgery, the patient presented to the emergency department with an asymptomatic subconjunctival hemorrhage in the OD. Examination revealed moderate punctate keratitis and a small painless paracentral epithelial defect with mild stromal ulceration, next to the site of perforation. After two weeks of treatment with aggressive lubrication, a bandage CL, antibiotics, and corticosteroids, the corneal ulcer showed no improvement (Figure 1 - A1, A2). Remarkably, at this moment, the OS, which had no previous findings, exhibited a painless translucent central corneal perforation without signs of inflammation (Figure 1 - B1, B2). Cyanoacrylate glue was immediately applied in the OS, along with antibiotics and a bandage CL (Figure 1 - B3, B4, B5), and the patient was scheduled for an amniotic membrane graft surgery. Concerned about the threat of imminent perforation of the corneal ulcer in the OD, topical insulin drops (4 i.d.; 1 U/mL) were initiated, along with a CL and topical fluoroquinolone. Beforehand, the patient was fully informed about the potential risks and benefits of off-label topical insulin use, and he provided informed consent. Also, after consulting with the assisting oncologist, further immunosuppression was not deemed appropriate, with the patient maintaining prednisolone 40 mg and cyclosporine 50 mg daily. Within three days of treatment, the OD corneal ulcer was completely re-epithelialized, and the patient continued on topical insulin with progressive tapering to twice per day without additional medication. Two months later, the patient exhibited significant improvement in OD punctate keratitis and OSDI questionnaire severity score (41.7, based on the same six questions as the initial assessment), with BCVA improving to 20/125 (Figure 1 - A3, A4, A5).

**Figure 2:**
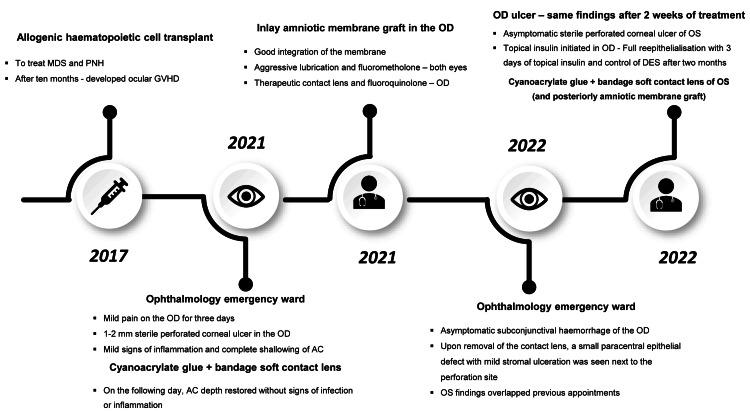
Timeline of the clinical case. AC, anterior chamber; DES, dry eye syndrome; GVHD, graft-versus-host disease; MDS, myelodysplastic syndrome; OD, right eye; OS, left eye; PNH, paroxysmal nocturnal hemoglobinuria.

Topical insulin was not initiated in the left eye (OS) due to incomplete integration of the amniotic membrane graft at the time of the last appointment. The patient was subsequently lost to follow-up. A timeline summarizing the case information is presented in Figure 2, and the main findings are further displayed in Table 1. 

**Table 1:** Summary of main findings at each time point for both eyes AC, anterior chamber; BCVA, best-corrected visual acuity; CL, contact lens; HM, hands motion; LP, light perception; OD, oculus dexter; OS, oculus sinister; OSDI, Ocular Surface Disease Index.

Time point	OD BCVA	OS BCVA	OD slit-lamp	OS slit-lamp	OSDI	Intervention OD	Intervention OS
Presentation	LP	20/400	Central perforation, shallowing of AC	Leukomas, mild keratitis	-	Cyanoacrylate glue + CL + amniotic graft + topical antibiotics	-
1 month post-OD surgery	-	-	Integration of the graft. Deep AC.	Similar findings	83.3	Artificial tears + fluorometholone	Artificial tears + fluorometholone
4 months post-OD surgery	HM	20/400	Subconjunctival hemorrhage + paracentral epithelial defect + mild ulceration	Similar findings	-	Artificial tears + CL + antibiotics + fluorometholone	Maintained therapy
2 weeks after conservative measures in OD	-	-	Refractory paracentral epithelial defect + mild ulceration	Translucent central corneal perforation	-	Topical insulin + CL + antibiotics	Cyanoacrylate glue + CL + amniotic graft + antibiotics
3 days post-insulin in OD	-	-	Complete re-epithelialization	Incomplete integration of amniotic graft	-	Topical insulin + CL + antibiotics	Maintained therapy
2M post-insulin in OD	20/125	HM	Significant improvement of keratitis, no epithelial defect	Incomplete integration of amniotic graft	41.7	Tapering of topical insulin drops	Maintained therapy

## Discussion

Ocular GVHD is a relatively common complication in patients undergoing allo-HSCT, affecting 40-60% of patients. Risk factors for oGVHD include male recipients of female donors, skin, oral mucosa, liver, lung, and gastrointestinal tract involvement. The incidence of severe dry eye in cGVHD is higher in recipients of peripheral blood stem cell transplantation or bone marrow transplantation compared to those receiving cord blood transplantation [1]. Standard treatment primarily consists of supportive care with topical lubricants, immunosuppressants, antibiotics, and CL. However, despite treatment, oGVHD may progress to PEDs and corneal ulcers, necessitating alternative approaches such as plasma-rich growth factors or amniotic membrane graft surgery [1,2]. In this case, topical insulin was utilized to treat a corneal ulcer associated with oGVHD and to control DES, which had not been achieved with previous treatments. While the role of topical insulin in promoting epithelial cell growth and migration in corneal ulcers is well established, its effectiveness in controlling oGVHD is not well established in the literature. We hypothesize that, in addition to its mitotic effects, topical insulin may inhibit epithelial cell autophagy through the phosphoinositide 3-kinase/Akt/mTOR pathway, thereby modulating the apoptotic environment of oGVHD and ameliorating its complications [3,7,8]. Additionally, insulin’s anti-inflammatory properties may play a role in mitigating GVHD by reducing corneal inflammation and restoring limbal homeostasis. This is particularly significant, as the inflammatory milieu, including chronic inflammatory cell infiltration and enzyme activity (CD68+ and CD8+ cells, matrix metalloproteinase 9), is thought to contribute to limbal stem cell deficiency and keratocyte apoptosis in oGVHD [1,2]. Recent data have shown that insulin inhibits the TLR4/NF-κB signaling pathway, leading to reduced expression of pro-inflammatory cytokines such as IL-1β and IL-6, thereby promoting corneal epithelial cell proliferation and migration during wound healing [9]. 

Ocular perforation, though rare (with an estimated incidence of <1.6%), is a severe consequence of uncontrolled oGVHD. A recent multicenter study outlined the clinical features of oGVHD patients who developed corneal perforation, reporting that all patients presented with a translucent ulcer without associated infiltrates and poor BCVA (20/150 or worse) [2]. Our patient’s findings align with this study, suggesting a common disease pathway and presentation. Despite this, the pathophysiology of corneal perforation in oGVHD remains poorly understood. Neurotrophic keratitis has been suggested as a potential contributor to this complication, possibly due to peripheral neuropathy associated with GVHD. This hypothesis is supported by our patient’s bilateral diminished corneal sensitivity and spontaneous perforation without significant symptoms [2,10]. In theory, longstanding oGVHD may lead to trigeminal neuropathy, which, combined with the inflammatory environment, exacerbates oGVHD, leading to recurrent hypoesthetic corneal ulcers and, eventually, ocular perforation. Indeed, a high prevalence of small-fiber neuropathy has been shown in patients with cGVHD, which supports the spontaneous and asymptomatic nature of corneal perforations observed in these cases [10]. 

While most oGVHD patients can maintain good visual acuity throughout their lives, the emergence of severe complications significantly worsens their prognosis. This underscores the importance of reports that highlight novel and effective strategies for controlling disease progression. Another article regarding the potential of topical insulin is available in the published literature. Tahmaz et al. included seven (two acute myeloid leukemia, two acute lymphoblastic leukemia, two chronic myeloid, and one chronic lymphoblastic leukemia) patients with GVHD and DES, who had previously undergone treatment with topical steroids, artificial tears, and serum eye drops 10x/day [5]. Following topical insulin 4 i.d. for a mean of 5.4 weeks, there was a non-significant improvement of BCVA (0.44 to 0.29 logMar), OSDI score (70.83 to 64.73), and a significant improvement of the corneal fluorescein staining (Oxford grading 3.08 to 2.5).

This is the second known report of topical insulin therapy in ocular GVHD and the first to document rapid epithelial healing following bilateral spontaneous corneal perforation. Our patient’s outcome with topical insulin generally aligns with the findings of Tahmaz et al., showing increased visual acuity, reduced fluorescein staining, greater improvement in the OSDI score, with a fast time to re-epithelialization (three days), albeit with a prolonged tapering regimen to minimize the risk of recurrence of the epithelial defect; caution when interpreting these results is warranted as the loss of patient to follow-up may hinder the long-term efficacy analysis of topical insulin in this case. Although the systemic steroid dose had been increased from 20 mg to 40 mg one month prior to topical insulin initiation, the corneal ulcer remained refractory at the time. Therefore, while systemic immunosuppression may have stabilized the underlying inflammatory milieu, the timing and rapidity of the epithelial response after insulin suggest a direct topical effect. Nonetheless, we acknowledge that systemic therapy may have contributed to the overall improvement and that causality cannot be definitively established. That said, while the corneal ulcer may have had residual healing potential, the complete re-epithelialization within three days following insulin initiation, after two weeks of non-response to conventional therapy, suggests a contributory, if not primary, role for topical insulin in this case. Future reports should include in vivo confocal microscopy and/or AS-OCT to assess the integrity of the epithelial barrier, and report standardized ocular surface scores, such as the Oxford or NEI scales, to enable more objective and comparable evaluations. 

## Conclusions

In summary, we present a rare case of oGVHD with diminished corneal sensitivity, complicated by spontaneous and painless bilateral ocular perforation, suggesting a potential role for peripheral trigeminal neuropathy in the disease's progression. Furthermore, this case adds to the scarce literature on the use of topical insulin for treating refractory corneal ulcers and DES in oGVHD, demonstrating its potential as a novel therapeutic approach in these challenging cases.
